# Items of General Interest

**Published:** 1916-03

**Authors:** 


### Items of General Interest.

Dr. J. E. Baldwin, of Dallas, was found guilty on five counts in the indictment of violating the Federal narcotic law.

The city health board of Fort Worth have voted unanimously for a publicity campaign for the establishment of an independent health department.

Dr. J. E. Baldwin, of Dallas, has been placed on trial in the United States District Court under indictment charged with violating the Harrison anti-narcotic law.

An announcement has been received of the death of Mr. William Price Haygee, President of the Katharmon Chemical Company, of St. Louis, Mo., on February 3d.

Frog skin was grafted into the chest of Mrs. Samuel Sport, of Des Moines, Iowa, and physicians say she will now recover from severe burns sustained more than a month ago.

A new and modern sanitarium has been opened in Coleman at the corner of College Avenue and San Saba Street. Dr. A. F. Upton, who recently moved to Coleman, will be in charge.

The county commissioners have re-elected Dr. T. J. Milner, of Greenville, as county health officer. Dr. Millner has been health officer of Hunt county for many years and has done excellent work.

Subscriptions to a $3000 fund for the erection of cheap bungalows, to house indigent consumptives who go to San Angelo, are being received. It is believed the money will soon be raised in full.

Dr. Kate Huse, of St. Louis, has been appointed resident physician of the State Girls' Training School at Gainesville. Dr. Huse has been studying similar institutions, and goes to Gainesville highly recommended.

Authority has been granted by the officials of the I. & G. N. whereby employes of the company and others suffering from personal injuries may be treated by the company physician without going away to the hospital.

---

A tour of Texas cities, commencing in Houston, will be made by Miss Ella Phillips Crandall, executive secretary of the national organization of public health nursing, New York, in the interest of health betterment.

---

With two tin spoons, a pocketknife and a piece of rubber tubing as his instruments, Dr. H. W. Daniel of the Elkins Hospital, Elkins, W. Va., performed an operation on a woman apparently dying of diphtheria and saved her life.

---

Dr. Bernard Fantus of the University of Illinois College of Medicine is working out a system of administering unpleasant medicine in the form of candy. He disguises nauseous doses in the form of delicious chocolate tablets.

---

*The University Medical,* for the current month, edited and published by the students and alumni of the State Medical College, is just off the press. Several special articles from prominent physicians are included in this number.

---

Hotel Dieu, Beaumont's new hospital, during the year 1915 cared for more than 1400 patients. Of these, 800 of the treatments were in surgical cases and 600 were medical cases. During the winter months from fifteen to forty persons were fed daily.

---

Experiments with a new light cure have been apparently successfully carried out at St. Bartholomew's Hospital, London. They have found the rays have produced excellent results in the treatment of skin diseases and have stimulated the repair of shrapnel wounds.

---

Besides supporting itself, the City Hospital of Austin is now paying revenue into the treasury of the city. Nine hundred and eighteen dollars received during the month of January was turned over to the city by Dr. S. A. Woolsey, who has charge of the hospital's management.

---

Dr. G. D. Fairbanks, surgeon of the United States Immigration Service, has been honored by the German University of Leipsic.

Dr. Fairbanks has been making a special study of pellagra, and at a recent meeting of the county medical association read a specially prepared paper dealing with this disease and its treatment. Dr. Fairbanks has received a card from Dr. I. H. Rille of the German institution, asking that a copy of this paper be sent him.

---

### Texas State Board of Health, Austin.

The State bacteriological and chemical labtoratory is open to any person in Texas who is not in a position to have examinations made by other laboratories.

Care should be exercised in collecting and preparing specimens. Many specimens reach the laboratory in such a condition that it is impossible to make an accurate examination. Many packages are mailed in such condition as to be dangerous to those who have to handle them. To mail such packages is a violation of the postal laws. Directions will be furnished anyone requesting information as to the proper manner of collecting specimens and wrapping them.

The following examinations are made:

1. Throat cultures for diphtheria.
2. Sputum for tuberculosis, pneumococcus, etc.
3. Cerebro-spinal fluid.
4. Pus and exudates.
5. Blood for syphilis, typhoid and malaria.
6. Feces for intestinal parasites, hookworms, tapeworms, etc.
7. Urine.
8. Water and sewage for bacteria.
9. Also blood of animals for anthrax.

The laboratory manufactures tyhoid vaccine matter and autogenous vaccine, free of cost.

The laboratory is maintained by the State, and its services are free to any citizen.

Address all communications to Dr. G. M. Graham, State Bacteriologist, Austin, Texas.

---

"Oh, mother," sobbed the young wife, "John doesn't trust me."

"Why, my child, what has he done?"

"Well, you know, I cooked my first dinner for him today, and he invited a friend to dine with him." The sobs broke afresh.

"And oh, mother, the man was a doctor!"—*Stray Stories.*

